# Drug use profile in outpatient children and adolescents in different Italian regions

**DOI:** 10.1186/1471-2431-13-46

**Published:** 2013-04-04

**Authors:** Daniele Piovani, Antonio Clavenna, Maurizio Bonati

**Affiliations:** 1 Department of Public Health, Laboratory for Mother and Child Health, IRCCS - Istituto di Ricerche Farmacologiche “Mario Negri”, Via Giuseppe La Masa 19, Milan, 20156, Italy

**Keywords:** Children, Adolescent, Drug utilisation, Prescription, Outpatient

## Abstract

**Background:**

Large differences exist in the prevalence rate of drugs prescribed to children and adolescents between and within countries. The aim of this study was to evaluate child and adolescent drug prescription patterns in Italy in an extra-hospital setting at the regional and Local Health Unit (LHU) levels.

**Methods:**

Data sources were three regional prescription databases. Data concerning the year 2008 were evaluated. A total of 3.3 million children and adolescents were included. Drug prevalence and prescription rates were evaluated at the regional and LHU levels. The correlation between mean latitude, average annual income, hospitalisation rate, number of paediatricians per 1,000 resident children, and prevalence rate was evaluated by LHU using a linear multiple regression analysis.

**Results:**

Large differences were found across Italian regions and LHUs. The mean prevalence rate was 56.4% (95% CI 56.3-56.5%; 51.2-65.4% among regions) and, at the LHU level, ranged from 43.1% to 70.0% (higher in the South). A total of 878 drugs were prescribed, 175 of which were shared by all LHUs. Amoxicillin clavulanate was the most used drug in all regions and in 31 of 33 LHUs. Amoxicillin was the drug with the highest variability in use between LHUs (9.1-52.1% of treated children). An inverse correlation was found between prevalence rate and both latitude (p < 0.0001) and average annual income (p = 0.0002).

**Conclusions:**

The use of drugs in children and adolescents is higher in southern Italy and is inversely related to latitude and average annual income. More efforts should be devoted to informing physicians, patients and policy makers in order to plan effective initiatives to improve the situation.

## Background

Children are widely exposed to drugs, with an average prevalence rate of 60% worldwide [1]. There is a large variability between countries. Prevalence rates range from 51% in Denmark to 70% in Greenland and prescription rates range from 0.8 in Norway to 3.2 in the United States [1]. The highest exposure is observed in preschoolers (75-90%), while the prevalence rate decreases in children > 6 years old in all countries. The peak prevalence rate varies from below 2 years old in the United States to 3–5 years old in Greenland and Italy [1]. The drugs most frequently prescribed to children are antibiotics (20–33% of total prescriptions), anti-asthmatics (10-25%), and analgesics (10-16%) [1].

Quantitative and qualitative differences in antibiotic exposure between countries are well documented. For example, an Italian child has, on average, a fourfold higher risk of being exposed to antibacterial drugs than an English child and a threefold higher risk than a Dutch child [2]. Cephalosporins, a second line treatment in most paediatric infections, are widely prescribed in Italy (39% of paediatric antibiotic prescriptions) while in the Netherlands and Denmark this class represents less than one percent of total antibiotic paediatric prescriptions [2].

There is heterogeneity also in children’s exposure to respiratory system drugs between different countries: prevalence rates range from 6.2% in Norway to 19% in Italy [3]. ß2-mimetics and inhaled steroids are the most frequently prescribed asthma drug classes and in Italy the use of inhaled steroids is threefold and fourfold higher than in UK and in the Netherlands, respectively [4].

Wide international variation exists also in the prevalence rates of other, less common, classes of drugs used for chronic treatment in children, such as anti-epileptic and anti-diabetic drugs [5,6]. Such heterogeneity in different parts of the world may reflect differences both in prescription habits and in prevalence of diseases.

Prescribing differences exist also within Italy: the overall prevalence rate for all prescribed drugs in children is 57.3% in the North and 68.3% in the South [7]. Notable differences have been reported also within regions. This is the case of the Lombardy region, where, in 2006, prevalence rates for all drugs ranged from 38.4% in Milan’s local health unit (LHU) to 54.8% in Brescia’s [8].

The literature suggests that the geographic factor is one of the most important determinants in drug prescription. In this context, a comparison between prescribing profiles at the LHU level was made, involving three prominent Italian regions: Lombardy, Lazio, and Puglia. The main goal of this study was to evaluate the influence of territorial setting on the drug prescription profile of a child and adolescent outpatient Italian population. The association between prevalence rates and a few socio-economic determinants was investigated.

## Methods

### The Italian national health service (NHS)

Italian healthcare is provided free or at a nominal charge through a network of LHUs. Every Italian resident is registered with a family (paediatric or general) practitioner. Children are assigned to a paediatrician until they are 6 years old; afterwards, the parents can choose to register a child with a general practitioner. All adolescents > 13 years old are assigned to a general practitioner. A national formulary is available, in which drugs are categorised into two classes: class A includes essential drugs that patients do not have to pay for and class C contains drugs not covered by the NHS (e.g. contraceptives, nonsteroidal anti-inflammatory drugs, dermatologic drugs).

### Data source

The data sources were regional databases routinely updated for administrative and reimbursement reasons. The databases stored all community (i.e. outside hospital) prescriptions reimbursed by the NHS. Aggregated and anonymous data were provided by the regional health ministry of Lombardy, Lazio and Puglia regions. No approval by an ethic committee was required. The study population was composed of 3,301,096 children and adolescents < 18 years old. It represented about one third of the population for that age group, living in three large Italian regions: Lombardy (1,616,268 resident children, North Italy), Lazio (926,015 children, Centre), and Puglia (758,813 children, South).

All drugs were classified according to the ATC system (Anatomical Therapeutic Chemical classification system). The observation period was from 1 January to 31 December 2008. A total of 33 LHUs were included in the study.

### Measures

The following measures were used in order to describe the prescription profile:

1) prevalence rate, expressed as the percentage of resident children/adolescents (<18 years old) receiving at least one prescription in one year.

2) prescription rate, expressed as number of prescriptions per treated children (every child/adolescent receiving at least one prescription in one year was considered a treated child). These measures were retrieved at the regional and LHU levels.

The percentage of treated children for the 10 most prescribed active substances was calculated at the regional and LHU levels.

The all-cause hospitalisation rate was calculated for each LHU, expressed as number of children with at least one hospital admission in one year divided by 1000 resident children/adolescents. The number of paediatricians per 1000 resident children was calculated for each LHU.

The number of active substances used at least once across the three regions and across LHUs were retrieved (shared drugs). The drugs shared by all LHUs of each region were compared with the WHO “List of essential medicines for children” [9], and with the British National Formulary for children (BNF for children) year 2011.

The ranks corresponding to the percentage of treated children for the 10 most prescribed active substances were compared by using the W Kendall concordance test (regional level).

The non-parametric Spearman test was used to evaluate the correlation between prevalence rates and the following determinants at the LHU level:

latitude,

hospitalisation rate for all causes,

number of paediatricians per 1000 resident children,

average annual income.

A stepwise linear multiple regression analysis was performed considering each LHU’s prevalence rate as the dependent variable group and the aforementioned four determinants as the independent variable groups (fixing α = 0.15).

The coefficient of variation (CV) was calculated as the ratio of the standard deviation (SD) to the mean for various measures. The Mantel-Haentzel *χ*^2^ test was performed in order to compare the drug prescription prevalence in boys and girls. A choropleth map of available prevalence rate data for LHUs was created using the software Arcmap version 10. The prevalence values were categorised into three classes calculated on the basis of the mean ± SD. Statistical analysis was performed using SAS software, version 9.1.

## Results

### Prescription profile

**Figure 1:**
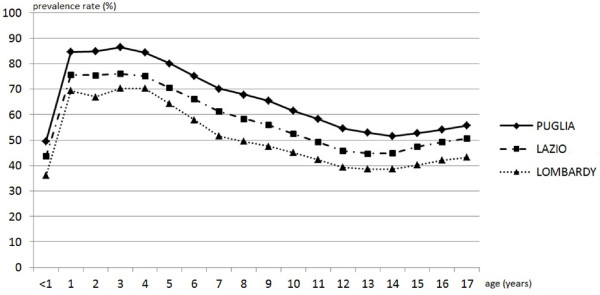
**Prevalence rate by age.** All drugs prevalence rate (%) by age (year) for each region.

During 2008, 1,861,425 children and adolescents (56.4% of the study population, 95% CI 56.3-56.5%) received at least one drug prescription. Prevalence and prescription rates by region are reported in Table 1. The prevalence rate of all drugs was slightly higher in boys than in girls for all ages (57.3 vs 55.4%; *χ*^2^ = 1261; p < 0.001). The male/female odds ratio for prevalence rate for all drugs was, on average, 1.08, with no statistically significant differences found between the three regions. The trend for gender was similar up to 15 years of age, after which the prevalence rate for girls was higher than that for boys. The profile curve of the prevalence rate by age was very similar in the three regions (Figure 1).

**Table 1:** Prevalence (%) and prescription rates (number of prescriptions/treated children) by regions

	**Prevalence rate**		**Prescription rate**	
Lombardy	51.2	(43.1-56.3)	3.3	(2.9-3.6)
Lazio	58.1	(49.9-68.1)	3.8	(3.4-4.4)
Puglia	65.4	(62.5-70.0)	4.2	(3.9-4.8)

Ranges by local health unit (LHU) are reported in brackets.

A total of 6,844,307 prescriptions were dispensed. Each treated child/adolescent received 3.7 ± 0.4 prescriptions. Boys received more prescriptions (3.8 vs. 3.5) than girls. Infants (<6 years old) treated with at least one drug received more prescriptions (4.3 prescriptions per treated individual) than children (3.3) and adolescents (3.1). The highest number of prescriptions/treated child was observed in 4 year old children (4.7).

### Prescription profile by LHU

**Figure 2:**
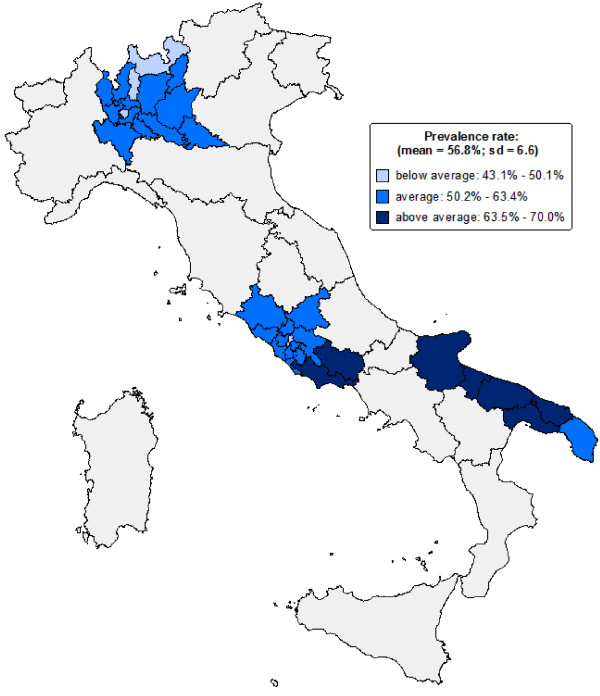
**Prevalence rate of drugs.** Choropleth map of regions and Local Health Units (LHUs) included in the study. The prevalence values were categorised into three classes calculated on the basis of the mean ± SD.

The prevalence rate for all drugs among all LHUs ranged from 43.1% to 70.0%, with a CV = 0.12 (Figure 2). The within-region CV among the different LHUs ranged from 0.04 in Puglia to 0.09 in Lazio.

The prescription rate ranged from 2.9 to 4.8 with a CV = 0.12. The within-region CV among the different LHUs ranged from 0.05 in the Lombardy Region to 0.09 in the Lazio Region.

### Determinants of prescriptions

Spearman’s tests found an inverse correlation between prevalence rate and latitude (r_S_ = −0.78; p < 0.0001) and between prevalence rate and average annual income (r_S_ = −0.81; p < 0.0001). Hospitalisation rate showed a weak correlation with prevalence rate (r_S_ = 0.49; p = 0.005).

The multiple linear regression model confirmed the inverse association of prevalence rates toward latitude (partial R-squared = 0.76; p < 0.0001) and average annual income (partial R-squared = 0.10; p = 0.0002), while the hospitalisation rate and number of paediatrician per 1000 resident children did not reach the minimum significance value.

### Active substances

A total of 878 different active substances were prescribed, ranging from 639 in Puglia to 800 in Lombardy. Of these active substances, 530 were shared by the three regions, and 175 by all the LHUs. The number of active substances shared by all the LHUs of a single region ranged from 190 in the Lombardy region to 340 in the Puglia region.

The drugs shared by all the LHUs belonged to 45 therapeutic main groups. The most represented were: antibacterials for systemic use (J01), with 35 active substances; and drugs for obstructive airway diseases (R03), with 13 active substances. Among the 35 antibiotics, the most represented classes were cephalosporins (9 active substances), and macrolides (8).

Of the drugs shared by all LHUs, 139 out of 175 (79%) were included in the BNF for children and 46 out of 175 (26%) were included in the WHO “List of essential medicines for children”. Of the 36 drugs prescribed in all the LHUs, but not included in the BNF for children, 12 were antibiotics. The 10 most prescribed active substances at the regional level comprised, on average, 65.5% of all prescriptions (from 62.4% in Puglia to 68.5% in Lombardy). The W Kendall test scored a concordance coefficient of 0.81 (p < 0.01). Ranks associated with each active substance are reported for each region in Table 2. Of the 10 most prescribed active substances, only 6 were in the top 10 in all the LHUs. Of these, the drug with the most heterogeneous prevalence rate was amoxicillin, with a rate ranging from 9.1% to 52.1% of treated children among all LHUs (CV = 0.47). The drug with the most homogeneous prevalence rate was amoxicillin clavulanate (CV = 0.10), covering 41.6% (33.8-52.1%) of treated children. Beclometasone was the most used respiratory-system drug and scored second in rank among all active substances, with 23.5% (12.5-30.2%) of treated children. Amoxicillin was the second most prescribed drug in the Lombardy region, while it was the sixth and seventh in the Puglia and Lazio regions. Ranks associated with the most prescribed drugs were not statistically different among the three age strata considered (infants, children, adolescents).

**Table 2:** List of the most 10 most used active substances in the population study and in the three regions ordered by % of treated children

	**% of treated children**			
	**Study population**	**Regions**		
		**Lombardy**	**Lazio**	**Puglia**
amoxicillin clavulanate	41.6	43.2 (1)	40.2 (1)	41.5 (1)
beclometasone	23.5	18.8 (3)	26.2 (2)	25.5 (2)
clarithromycin	18.5	14.3 (5)	18.8 (3)	22.5 (3)
amoxicillin	18.2	27.7 (2)	13.3 (7)	13.7 (6)
salbutamol	14.8	15.2 (4)	17.9 (4)	11.2 (7)
cefixime	13.8	6.6 (9)	15.7 (5)	19.2 (4)
azithromycin	12.8	10.4 (6)	12.8 (8)	15.2 (5)
betametasone	9.7	7.2 (8)	13.6 (6)	8.3 (9)
cefaclor	8.0	7.5 (7)	9.2 (9)	7.2 (11)
cetirizine	5.9	5.8 (10)	6.6 (11)	5.4 (15)
	0-5 years old	Lombardy	Lazio	Puglia
amoxicillin clavulanate	48.6	49.5 (1)	46.1 (1)	49.9 (1)
beclometasone	31.5	25.9 (3)	36.4 (2)	36.7 (2)
amoxicillin	27.5	37.0 (2)	19.4 (5)	18.4 (5)
salbutamol	22.4	21.6 (4)	27.1 (3)	18.1 (6)
clarithromycin	20.9	14.6 (5)	23.6 (4)	30.1 (3)
cefixime	13.5	6.7 (11)	18.4 (7)	21.2 (4)
betametasone	13.5	11.8 (6)	18.6 (6)	10.8 (12)
azithromycin	12.8	9.8 (8)	13.6 (8)	17.9 (7)
cefaclor	11.7	11.5 (7)	12.7 (9)	11.0 (10)
flunisolide	7.9	7.7 (9)	7.8 (11)	8.9 (14)
	6-11 years old	Lombardy	Lazio	Puglia
amoxicillin clavulanate	41.7	42.6 (1)	40.9 (1)	41.1 (1)
beclometasone	19.8	15.8 (3)	22.8 (2)	23.0 (2)
clarithromycin	16.2	14.2 (4)	17.0 (3)	18.7 (4)
amoxicillin	16.2	23.3 (2)	9.9 (9)	11.3 (6)
cefixime	12.8	6.6 (8)	15.5 (4)	19.8 (3)
azithromycin	12.7	10.8 (6)	12.8 (6)	15.5 (5)
salbutamol	12.5	12.5 (5)	15.4 (5)	9.4 (7)
betametasone	8.1	5.3 (10)	11.7 (7)	8.6 (8)
cefaclor	7.8	6.6 (9)	10.0 (8)	7.6 (10)
cetirizine	7.5	7.7 (7)	8.1 (10)	6.8 (12)
	12-17 years old	Lombardy	Lazio	Puglia
amoxicillin clavulanate	31.9	33.2 (1)	30.5 (1)	31.5 (1)
clarithromycin	14.8	13.7 (3)	13.8 (3)	17.5 (2)
beclometasone	12.8	10.2 (5)	15.0 (2)	14.4 (4)
amoxicillin	12.6	17.2 (2)	8.2 (7)	10.5 (6)
azithromycin	11.3	11.0 (4)	11.7 (5)	11.4 (5)
cefixime	10.9	6.4 (8)	12.0 (4)	16.0 (3)
salbutamol	6.5	7.5 (6)	7.3 (8)	4.5 (9)
cetirizine	5.6	6.5 (7)	5.3 (9)	4.7 (8)
betametasone	4.7	1.8 (15)	8.3 (6)	5.1 (7)
levocetirizine	4.6	4.5 (9)	5.0 (10)	4.3 (10)

The numbers in brackets represent the rank corresponding to each active substance in each region.

## Discussion

This study provides an analysis of the prevalence of drug use in children and adolescents in three large Italian regions with different socio-economical and geographical characteristics. About one third of the child/adolescent Italian population was included.

The prevalence was heterogeneous across the different regions and even more so across LHUs. The Lazio region had the highest within-region variability, while the Lombardy and Puglia regions had, respectively, the lowest and the highest prevalence rates. The linear regression model found a strong inverse correlation between prevalence rates and latitude, and between prevalence and average annual income. The average annual income (per-capita) in southern areas of Italy is lower than in northern ones (19,541 € in Puglia vs 25,488 € in Lombardy) and drug use in children and adolescents is higher in these regions and LHUs. Some evidence of similar findings is available from other studies, [10,11] but these were not related to outpatient children and adolescents. The regression model did not find relevant correlation between prevalence rates and hospitalisations for all causes (as moderate/severe “proxy of disease”) indicating that an increased use of drugs in children, in general, is not closely related with a higher disease prevalence.

There are other factors which we could not investigate in our study such as the paediatricians attitude to prescription and the perceived parental expectation of a prescription which are two important factors influencing ambulatory antibiotic prescriptions to children in Italy [12,13]. Other studies found that both low educational and socioeconomic status of parents were associated with a higher risk of non-adherence to physician indications in antibiotic use [14,15] causing a higher rate of treatment failure and thus the need of a new prescription. Out of pocket drug prescriptions, which are not detectable in our database, has been associated to a higher socioeconomic and educational status of patients [14], however the phenomena is probably limited for outpatient children and adolescents.

An impressively large amount of drugs was prescribed. It is interesting to note that 40% of the 878 drugs prescribed was not shared at the regional level, and that only 20% was used at least once in all 33 LHUs. The list of drugs shared by all the LHUs (175 drugs) could be considered “essential”, independently of geographical setting. Nevertheless, redundancies also exist within this list. This is particularly evident when antibiotics, a class that covered 21% of the list, are considered. A low overlap (26%) between this list of drugs and the WHO “List of essential medicines for children” is in part expected, since this latter represents a minimal list of medicinal for priority conditions and for a basic health care system. It is notable, however, that three quarters of these shared drugs are not considered “essential” from a public health point of view. In all, 20% of the shared drugs were not reported in the BNF for children, despite the fact that the BNF is considered a reference formulary at the international level. In order to promote a more homogeneous and rational use of drugs in children at the national level, the adoption of a common, acknowledged European formulary of paediatric medicines should be considered [16].

The most widely used drug was amoxicillin clavulanate. Most Italian paediatricians still consider it as the first choice drug for paediatric infectious diseases, as already documented in the 0–14 year old Italian population [17]. Despite the fact that amoxicillin clavulanate has a wider activity spectrum than amoxicillin alone, toxicity is higher and the cost effectiveness (for infections caused by non-β-lactamase producing bacteria) is lower [18,19].

Amoxicillin was the active substance with the highest variability in use between the different LHUs: there was almost a six fold higher consumption between LHUs ranked as the first and the last. Amoxicillin should be the most widely used drug. National and international guidelines consider it the first line treatment for acute respiratory infections (in particular pharyngotonsillitis and acute otitis media) [20-28], which are the most prevalent diseases in children. It is a major concern that in Lazio and Puglia amoxicillin was, respectively, the seventh and the sixth most used drug. In Lazio school-aged children amoxicillin was only the ninth most used drug. No data on paediatric resistance in outpatients are available at the regional level, but it is unlikely, although possible, that an increased prevalence of β-lactamase producing bacteria in the South could be responsible for such macroscopic differences. In the LHU with the highest consumption of amoxicillin (Lombardy Region), a training program for paediatricians, based on the main international guidelines on paediatric infectious diseases, [29] has been running since 2004. The data from the current study confirms that such interventions can have a large impact on prescription habits.

Beclometasone was the second most prescribed drug in the study population. In Italy beclometasone is prescribed mainly as symptomatic treatment of upper respiratory tract infections and is quite never prescribed for asthma therapy [30,31]. Beclometasone was more prescribed in the South, suggesting that contexts with a higher prevalence of drug prescriptions are characterised by greater inappropriateness.

Overall findings suggest that reported national drug prescription rates should be viewed with criticism if within country differences are not considered or reported.

### Strengths and limits

To date this is the largest study looking at drug prescriptions in Italian children and adolescents.

The observed prevalence rate was similar compared to what documented in a previous study including 22 Italian LHUs [7]. In comparison with the previous study we were able to include all the LHUs of three large regions representative of different economic, socio-demographic, and geographic settings, allowing the comparison of the different regional prescription profiles. The current study also included the analysis of other determinants of prescriptions and the analysis of the number of active substances shared by regions and LHUs.

Nevertheless, there are some limitations. Information on the disease was lacking, but this is an intrinsic limitation of all pharmacoepidemiology studies using prescription databases. Data concerning the use of private paediatric practice across the three regions was not available. Differences in the use of private practice could potentially influence the different prescription patterns observed in our study since only reimbursed prescriptions are detectable in our database. Other data, however, suggest that the out-of pocket drug use may be limited for reimbursed drugs [32].

We did not include data from a random sample of the Italian population (because such data were not available for all regions/LHUs), thus, from a strictly statistical point of view, our results may not be valid for all Italian regions. However, we included three large regions (from North, Centre, and South of Italy, which represent almost 1/3 of the Italian child population) which are quite representative of the differences in socio-economic status found in Italy. Moreover, the fact that drug consumption is higher in southern Italy is reported in the annual report conducted by OsMed [33] (*Osservatorio sull’impiego dei medicinali*) in the general population, thus it is very likely that this trend is maintained in children.

## Conclusions

In conclusion, this is the first study that compares the complete drug prescription profile in outpatient children of three of the most prominent Italian regions.

The data indicates that Italian prevalence rate of drugs in children and adolescents is similar to that observed in other countries in Europe, but there is a very high prevalence rate in southern Italy. The average annual income was inversely related to drug prescriptions, recalling a well-known North–South gap. Focused studies are warranted to clarify the role of other determinants or possible confounders such as socio-cultural factors, physicians prescribing attitude, the use of private practice, out-of-pocket drug use, marketing strategies and medication adherence across the different areas. More efforts should be devoted to informing physicians and patients, as well as policy makers, of such a scantly rational use of drugs in Italian children while waiting for effective initiatives aimed at improving the situation.

## Competing interests

The authors declare that they have no competing interests.

## Authors’ contributions

All the authors contributed equally to the design of the study. DP collected the data, undertook the statistical analysis and wrote the first draft of the manuscript. AC contributed in planning the data analysis and in writing the manuscript. AC and MB supervised the study. All authors contributed to and have approved the final manuscript.

## Interregional Italian drug utilisation group

Lombardy Region: Angela Bortolotti, Ida Fortino, Luca Merlino

Lazio Region: Marina Davoli, Ursula Kirchmayer

Puglia Region: Ambrogio Aquilino, Francesco Bux

Department of Clinical Pharmacology and Epidemiology, Consorzio Mario Negri Sud: Antonio D’Ettorre, Vito Lepore

## Pre-publication history

The pre-publication history for this paper can be accessed here:

http://www.biomedcentral.com/1471-2431/13/46/prepub
